# Diagnostic accuracy of circulating tumor cells detection in gastric cancer: systematic review and meta-analysis

**DOI:** 10.1186/1471-2407-13-314

**Published:** 2013-06-27

**Authors:** Lanhua Tang, Shushan Zhao, Wei Liu, Nicholas F Parchim, Jin Huang, Youhong Tang, Pingping Gan, Meizuo Zhong

**Affiliations:** 1 Department of Oncology, Xiangya Hospital, Central South University, Changsha, Hunan, China; 2 Eight-Year Program, Xiangya Hospital, Central South University, Changsha, Hunan, China; 3 Medical School/Graduate School of Biomedical Sciences, University of Texas Health Science Center, Houston, Texas, USA

**Keywords:** Circulating Tumor Cells (CTCs), Gastric Cancer, Meta-analysis, Diagnostic Accuracy

## Abstract

**Background:**

Circulating tumor cells (CTCs) detection has previously been used for diagnosing gastric cancer. However, the previous studies failed to make an agreement whether the detection of CTCs contributes to the diagnosis of gastric cancer.

**Methods:**

A systematic review and meta-analysis was performed to evaluate the overall accuracy of CTCs detection for diagnosing gastric cancer. PubMed, Embase and the Wanfang database were searched in all languages published up to Oct 2012. The pooled sensitivity (SEN), specificity (SPE), positive and negative likelihood ratios (PLR and NLR, respectively), diagnostic odds ratio (DOR) and summary receiver operating characteristic (sROC) curve were calculated to evaluate the overall test performance.

**Results:**

Twenty studies were included in this systematic review and meta-analysis. The diagnostic value of CTCs detection for the gastric cancer was calculated to evaluate the overall test performance. The summary estimates of The pooled sensitivity, specificity, positive and negative likelihood ratios, diagnostic odds ratio were 0.42 (95% confidence interval (CI), 0.21-0.67), 0.99 (95% CI, 0.96-1.00), 58.2 (95% CI, 9.8-345.9), 0.58 (95% CI, 0.38-0.89), and 100 (95% CI, 15–663), respectively. The summary receiver operating characteristic curve was 0.97 (95% CI, 0.95–0.98). Deek’s funnel plot asymmetry test found no evidence of study publication bias in the current study (P = 0.49).

**Conclusion:**

This systematic review suggests that CTCs detection alone cannot be recommended as a screening test for gastric cancer. However, it might be used as a noninvasive method for the confirmation of the gastric cancer diagnosis.

## Background

Gastric cancer is the 4th most frequently diagnosed cancer and the second leading cause of cancer-related death [1]. It was estimated that 989,000 new cases and 738,000 deaths had occurred worldwide in 2008 alone, which accounted for 8 percent of the total new cases and 10 percent of the total deaths [2]. Globally, gastric cancer rates were about twice as high in males as in females. The highest gastric cancer incidence rates were reported in Eastern Asia, Eastern Europe, and South America and the lowest rates in North America and most parts of Africa [3].

Generally, the current routine of the diagnosis is based on symptoms, signs, serum tests of tumor markers, radiology, and pathology. Unfortunately, most patients have advanced gastric cancer at the time of diagnosis [4]. The more advanced the tumor is, the worse the prognosis [5]. The five-year survival rate for advanced gastric cancer patients is 3.1% (1,4 in survival of metastatic gastric cancer significant of age, sex), while the 5-year survival of patients with early gastric cancer is over 90% (3 in prognostic factors in advanced gastric cancer). Although great improvements have been made recently in the treatment of gastric cancer, the high incidence of metastasis and recurrence continue to affect the clinical management [6]. To improve the clinical outcomes of patients with gastric cancer, new methods and techniques were developed to facilitate the diagnosis of this disease.

Circulating tumor cells (CTCs) were first found in the peripheral blood of cancer patients in 1869 [7], and they were defined as tumor cells originating from either primary or metastatic tumors and circulating in the peripheral blood [8,9]. During the initial phase of the micrometastasis, CTCs are shed intermittently from the solid tumors into the peripheral blood [10]. Then because of the blood mechanical shear forces, immune surveillance, and so on, most of CTCs will die, while a few remaining CTCs survive and then circulate successfully in the bloodstream, and later develop into clinically undetectable micrometastatic foci, which potentially grow into clinically apparent metastases [11].

During the past few decades, a variety of approaches to detecting CTCs have been developed. Generally, all the methods consist of two phases: enrichment or isolation/detection. The former includes morphologic-based isolation and immunological isolation, such as: isolation by size of epithelial tumor cells (ISET) [12,13], density gradient separation (Ficoll-Hypaque [14]), CTC-chip [15], microvortex-generating herringbone-chip [16], and so on. While the latter includes nucleic acid-based methods (PCR) and cytometric-based methods (flow cytometry) [17]. Besides, the CellSearch system, an enrichment and detection system, is the only approach approved by the US Food and Drug Administration (FDA) [18].

CTCs are reported to have the potential in assisting the diagnosis of gastric cancer [19,20], evaluating prognosis [21,22], monitoring the response of anticancer therapy and monitoring the early microstasis [4]. However, the current studies failed to reach an agreement in whether the detection of CTCs has contributed to the diagnosis of gastric cancer. So the diagnostic value of CTCs detection in gastric cancer was evaluated by the meta-analysis and systematic review.

## Methods

### Literature search

This meta-analysis was conducted according to guidelines for diagnostic meta-analysis [23,24]. PubMed, Embase and the Wanfang database were searched in Oct 2012 using the strategy of (circulating tumor cell OR circulating tumor cells OR CTC or CTCs OR isolated/circulating/disseminated tumor cells OR ITC) AND (Gastric cancer or Gastric Neoplasms or Stomach Cancer) without time or language restrictions. The references of the included studies were also searched manually to identify additional eligible studies.

### Inclusion and exclusion criteria

The inclusion criteria for this meta-analysis were: 1) studies about the diagnosis of gastric cancer with CTCs detection; 2) studies with raw data that true-positive, false-positive, false-negative and true-negative could be found or calculated; 3) studies with reference standard for the diagnosis of gastric cancer; 4) studies with more than 20 patients. Exclusion criteria were: 1) studies with duplicate data reported in other studies; 2) studies that were letters, editorials, case reports or case series.

### Data extraction and quality assessment

The two investigators (Lanhua Tang, Shushan Zhao) independently reviewed the titles and abstracts of all the records searched above, and excluded the reviews, editorials, letters, case reports or case series, and studies without direct link to the main subject. For records which could not be evaluated through the titles and abstracts, full texts were retrieved for detailed evaluation according to the inclusion and exclusion criteria. Disagreements were resolved by discussion with the senior investigator (Meizuo Zhong). The reasons why studies were excluded were listed.

Two reviewers independently extracted data from all the eligible studies: 1) basic characteristics of studies including name of the first author, year of the publication, country of origin, markers of CTCs detection methods, mean/median age, diagnosis criteria of gastric cancer, tumor stage distribution of patients, source of control; 2) methods of studies including study design, methods of the inclusion of patients and controls, methods of CTCs detection, the blood volume, time and methods of sample collection; 3) outcomes including the number of patients with true or false positive and true or false negative results, detection SEN. If the data of the results were not directly reported, they were calculated based on SEN and SPE or positive and negative predictive value. Disagreements were resolved by discussion and consultation with the senior investigator (Meizuo Zhong).

Subsequently, the two independent authors evaluated the quality of the studies by Quality Assessment of Diagnostic Accuracy Studies-2 (QUADAS-2) [25] and Standards for Reporting of Diagnostic Accuracy (STARD) [26].

### Data analysis

This systematic review and meta-analysis about the diagnostic accuracy of CTCs detection in gastric cancer was performed using Stata software (version 12.0, College Station, TX) with the MIDAS and METANDI modules and RevMan (version 5.1).

With regards to Stata software, continuity correction was implemented by an addition of 1 to avoid the trouble that the cells containing zero values might bring to the analysis process. And when a study adopted several markers for the CTCs detection, the marker with the best SPE or the best SEN was used for the analysis of the pooled diagnostic accuracy.

By using a bivariate regression approach, the summary receiver operating characteristic (sROC) curve was constructed. The area under the sROC curve was an alternative global measure of test performance. The pooled estimates of SEN and SPE were calculated as the main outcome measures. Meanwhile, the summary positive and negative likelihood ratios (pooled PLR and pooled NLR, respectively, defined as the ratio of the probabilities that the CTCs detection will be positive/negative in cases with gastric cancer versus those without gastric cancer) were also calculated. The value of pooled PLR higher than 10 indicate that the positive result of the given test is useful for the confirmation of presence of gastric cancer, while the value of pooled NLR lower than 0.1 indicate that the negative result is useful for the exclusion of the disease [27]. As a single indicator measure of the diagnostic test accuracy that comprises a combination of SEN and SPE [28], the diagnostic odds ratio (DOR) describes the odds of positive test results in patients with gastric cancer compared with the odds of positive results in those without the disease. It’s calculated as: DOR = PLR/NLR.

The between-study heterogeneity was evaluated by Q test and I-square statistics. The former indicates whether the heterogeneity is significant. An inconsistency index of 0% and *P* value of 0.05 and more indicate no observed heterogeneity, when I^2^ becomes higher, the heterogeneity becomes greater. And I^2^ values ≥50% indicates substantial heterogeneity, in this circumstance, the DerSimonian Laird method was applied for pooled analyses [29,30].

Furthermore, to explore the sources of between-study heterogeneity, a meta-regression was used according to the characteristics of the included studies. Subgroup analyses were also performed.

Publication bias was studied too by a regression of diagnostic log odds ratio against 1/sqn’t. A non-zero slope coefficient suggestive of significant small study bias (p value < 0.10) [31].

## Results

### Literature search

**Figure 1:**
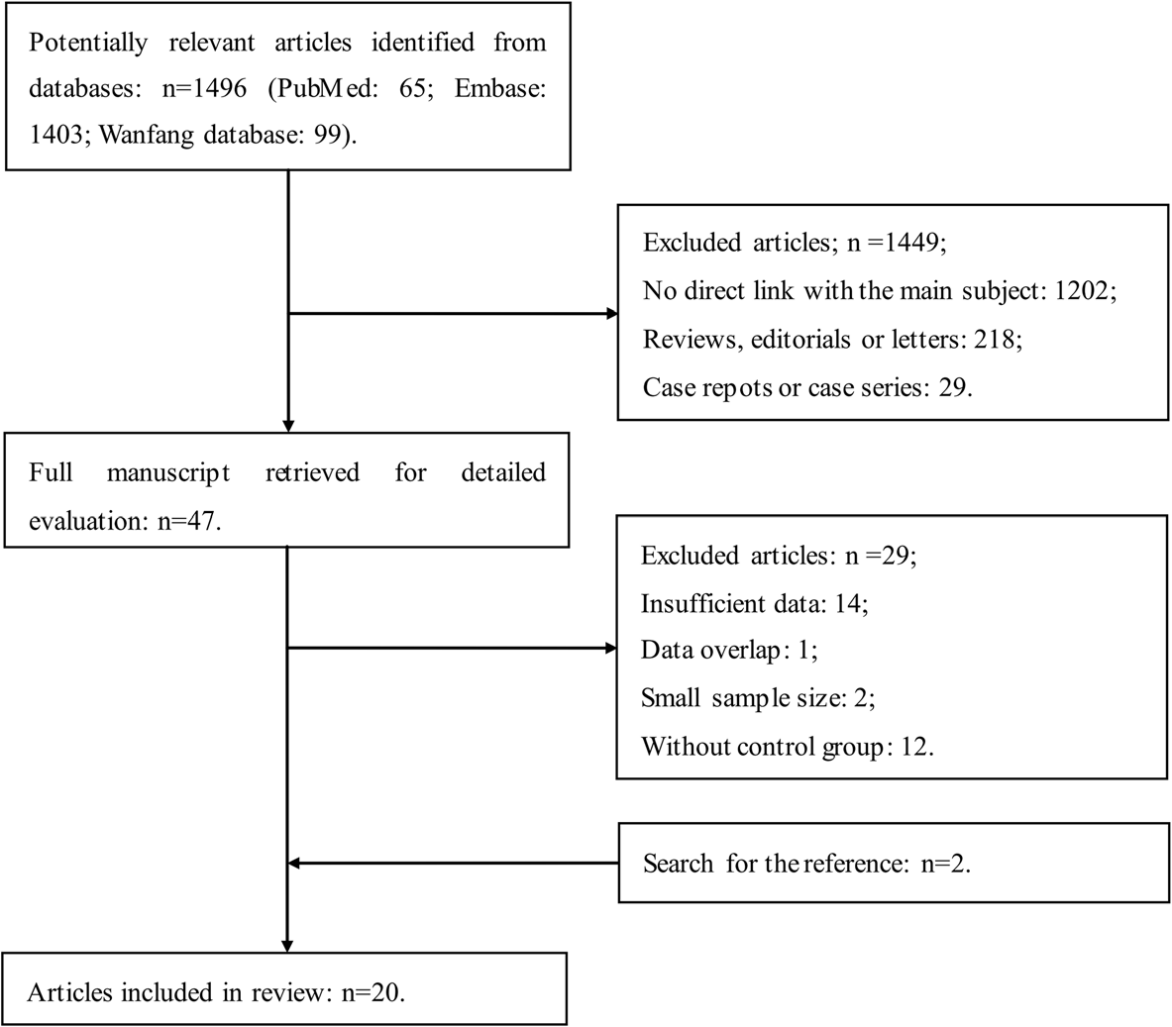
Flow diagram of study selection process.

The results of the literature research were presented in Figure 1. The initial search yielded a total of 1496 potential relevant studies. After the review of titles and abstracts, 1449 articles were excluded: 1202 articles had no direct link with the main subject; 218 of them were reviews, editorials or letters; and 29 were case reports or case series. Then 47 full manuscripts were retrieved for detailed evaluation. Finally, 20 studies [19-22,32-47] including a conference abstract [35] were included according to the inclusion and exclusion criteria. The remaining 29 studies were excluded because of the lack of sufficient data (n = 14), duplicate publications (n = 1), without control group (n = 12), and studies less than 20 patients (n = 2).

### Baseline characteristics

The main characteristics of the studies included in the meta-analysis were shown in Table 1.

**Table 1:** Main characteristics of studies included in the meta-analysis of the diagnostic accuracy of CTCs detection in gastric cancer

**First author**	**Year of publication**	**Country of origin**	**Maker used**	**CTC/patients**	**CTC/controls**	**tp**	**fp**	**fn**	**tn**	**Patient age(years) mean(range)**	**Tumor histology**	**Tumor stage**	**Data about prognosis**	**Inclusion criteria**	**Detection method**
Aihara	1997	Japan	Keratin 19	0/49	0/50	0	0	49	50	NR	NR	I-IV	No	UICC	RT-PCR
Bertazza	2009	Italy	Survivin	69/70	0/20	69	0	1	20	68(28–90)†	Yes	I-IV	Yes	UICC	qRT-PCR
			CK19	68/70	0/20	68	0	2	20						
			CEA	30/70	0/20	30	0	40	20						
			VEGF	27/70	0/20	27	0	43	20						
Cui	2011	China	piR-651	66/93	6/32	66	6	27	26	preoperative:60 ± 17; postoperative:63 ± 14	Yes	I-IV	Np	National Comprehensive Cancer Network clinical practice guideline of oncology	qRT-PCR
			piR-823	75/93	6/32	75	6	18	26						
Hiraiwa	2008	Japan	EpCAM	17/41	0/41	17	0	24	41	NR	Yes	I-IV	Yes	AJCC	Immunological
Ikeguchi	2005	Japan	CEA	0/59	0/15	0	0	59	15	66.3(26–86)	Yes	I-IV	Yes	Japanese Classification of Gastric Carcinoma	RT-PCR
Ikeguchi	2003	Japan	CEA	1/55	0/40	1	0	54	40	65.4	Yes	I-IV	No	Japanese Classification of Gastric Carcinoma	RT-PCR
			CK19	0/55	0/40	0	0	55	40						
			CK20	15/55	2/40	15	2	40	38						
Ito	2010	Japan	GFP	27/27	0/80	27	0	0	80	56.1(39–76)	Yes	I-IV	No	AJCC	Immunological
Koga	2008	Japan	CK19	8/69	0/14	8	0	61	14	65.7	Yes	I-IV	No	Japanese Classification of Gastric Carcinoma	qRT-PCR
			CK20	10/69	0/14	10	0	59	14						
Majima	2000	Japan	CK19	5/52	0/14	5	0	47	14	NR	NR	I-IV	Yes	Creteria of the UICC	RT-PCR
			CK20	5/52	1/14	5	1	47	13						
Noh	1999	Korea	CEA	16/35	0/9	16	0	19	9	54.5(26–71)	Yes	I/III/IV	No	AJCC	RT-PCR
Qiao	2007	China	CK20	9/40	0/20	9	0	31	20	62.2	Yes		No	NR	RT-PCR
Ren	2011	China	EpCAM	20/33	0/60	20	0	13	60	NR	Yes	I-IV	No	AJCC	Immunological
Uen	2006	China	c-Met	32/52	2/36	32	2	20	34	60.0(34–84)	Yes	I-IV	Yes	AJCC	RT-PCR
			MUC1	37/52	3/36	37	3	15	33						
Wang	2009	China	MAGE-1	19/40	0/20	19	0	21	20	55.7(27–77)	Yes	I-IV	No	AJCC	RT-PCR
			MAGE-3	10/40	0/20	10	0	30	20						
Wu	2006	China	hTERT	52/64	14/80	52	14	12	66	60.5(36–84)	Yes	I-IV	Yes	AJCC	RT-PCR
			CK19	50/64	12/80	50	12	14	68						
			CEA	53/64	19/80	53	19	11	61						
			MUC1	54/64	13/80	54	13	10	67						
Wu	2006	China	hTERT	26/42	0/30	26	0	16	30	60.2(34–84)	Yes	I-IV	Yes	AJCC	RT-PCR
			CK19	29/42	1/30	29	1	13	29						
			CK20	26/42	1/30	26	1	16	29						
			CEA	33/42	0/30	33	0	9	30						
Yang	2002	China	CEA	24/40	1/34	24	1	16	33	51.2(38–76)	Yes	I-IV	No	AJCC	RT-PCR
Yeh	1998	China	CK19	7/34	0/33	7	0	27	33	57(31–81)†	Yes	I-IV	Yes	UICC	RT-PCR
Zhang	2007	China	CEA	4/45	0/13	4	0	41	13	60.5(42–78)	Yes	I-IV	No	UICC	RT-PCR
Zhou	2010	China	miR-106a	43/90	3/27	43	3	47	24	male: 62.3; female:59.2	Yes	NR	No	UICC	RT-PCR
			MiR-17	47/90	2/27	47	2	43	25						

†: median (range) of patient age (years).

A total of 1030 patients and 668 controls were included in this meta-analysis. The included studies were mainly performed in Asia (China: 55%, Japan: 35%, Korea: 5%), and the remaining one was conducted in Italy [22]. There are 5 articles in Chinese (25%), and the other 15 were in English. All but two studies [38,47] included patients of I-IV stage, whereas Noh *et al.*[38] did not included patients of stage II, and Zhou *et al.*[47] did not report the tumor stage.

There were 15 of 20 (75%) studies having peripheral blood samples collected before any treatments, while 3 [20,32,47] of 20 (15%) collected blood samples after the treatments in partial patients and 2 [40,45] did not report the time of sample collection. In order to avoid contamination by epithelial cells, 8 studies (40%) collected two consecutive blood samples, and only the second tube was used for analysis with the first tube discarded. Mean volume of the blood samples was 6.23 (range: 2–14) milliliter (ml) with 13 studies (65%) collecting ≤7.5 ml blood samples.

As for CTCs enrichment, 4 (20%) studies used density gradient separation (3 for Ficoll-Hypaque centrifugation method), 5 (25%) studies applied the acid guanidium-phenol-chloroform or (acid) guanidium thiocyanate-phenol-chloroform method, 6 (30%) studies adopted the RNeasy Mini Kits or QIAamp RNA blood Mini Kit extraction, 2 (10%) studies used immunomagnetic isolation, and 2 (10%) studies used lymphocyte separation medium. There was 1 (5%) study that did not report the cell enrichment method.

Polymerase chain reaction (PCR) based methods were applied in 17 (85%) of 20 studies to detect CTCs, among which reverse transcription or real time polymerase chain reaction (RT-PCR) was the most common method (11 of 20), 3 used quantitative RT-PCR (qRT-PCR), 2 used multiplex RT-PCR, and 1 adopted Nested PCR. Besides, there were 2 (10%) studies adopted immunological methods, and 1 (5%) used the CellSearch system. The most frequently used markers of PCR-based methods were carcinoembryonic antigen (CEA, evaluated in 8 of 20 studies, 40%) and cytokeratin-19 (CK-19, evaluated in 8 of 20 studies, 40%) followed by cytokeratin-20 (CK-20, evaluated in 5 of 20 studies, 25%), other markers were EpCAM (10%), hTERT (10%), MUC1 (10%), c-Met (5%), MAGE-1 (5%), Survivin (5%), VEGF (5%), MAGE-3 (5%), GFP (5%).

### Assessment of study quality

**Figure 2:**
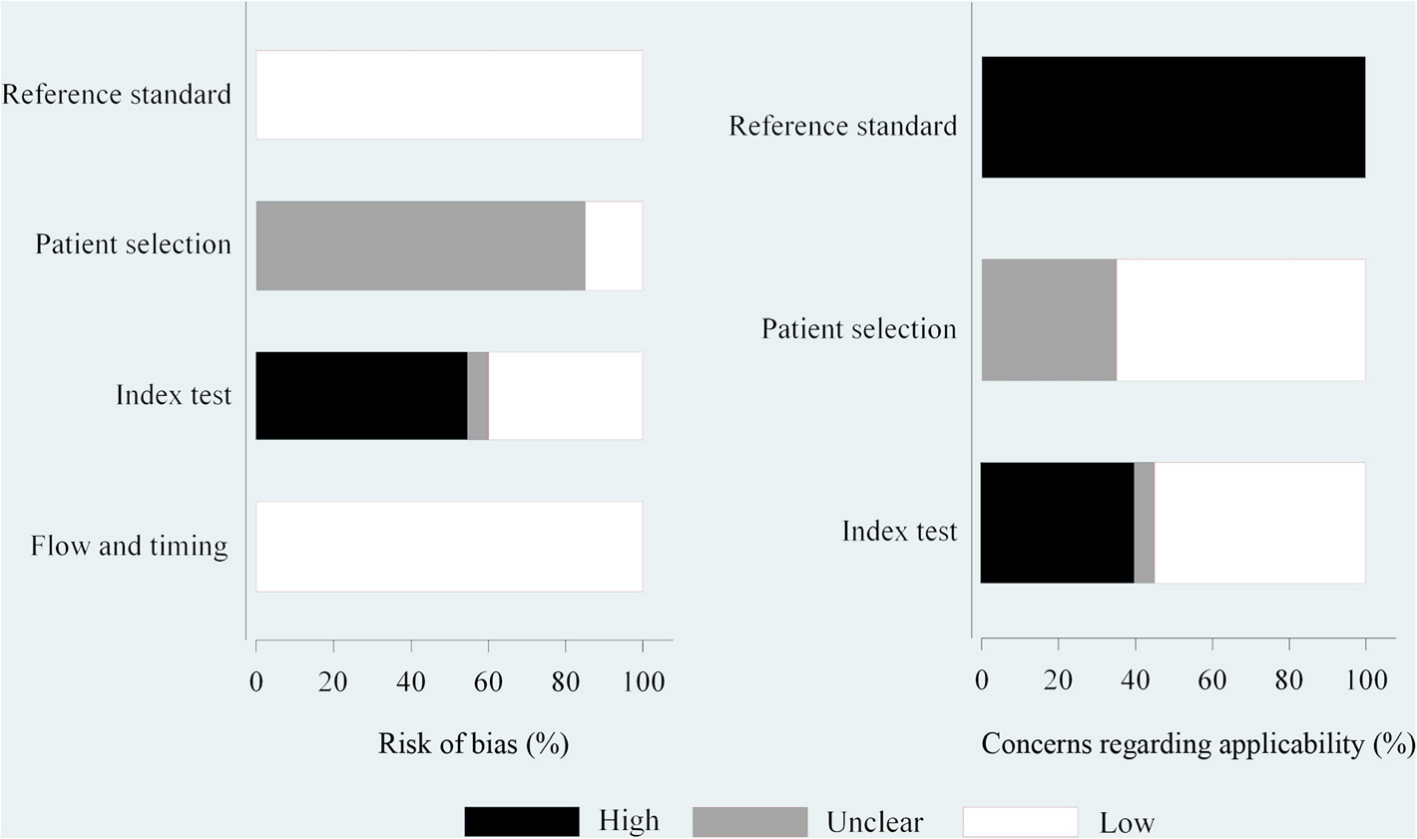
Overall quality assessment of included studies (QUADAS-2 tool): proportion of studies with low, high, or unclear risk of bias (left), proportion of studies with low, high, or unclear concerns regarding applicability (right).

Quality assessment was shown with a bar graph according to the QUADAS-2 tool in Figure 2. 11 of 20 studies in this meta-analysis fulfilled 18 or more of the 25 items in the STARD (Additional file 1: Table S1).

### Diagnostic accuracy of CTCs detection

**Figure 3:**
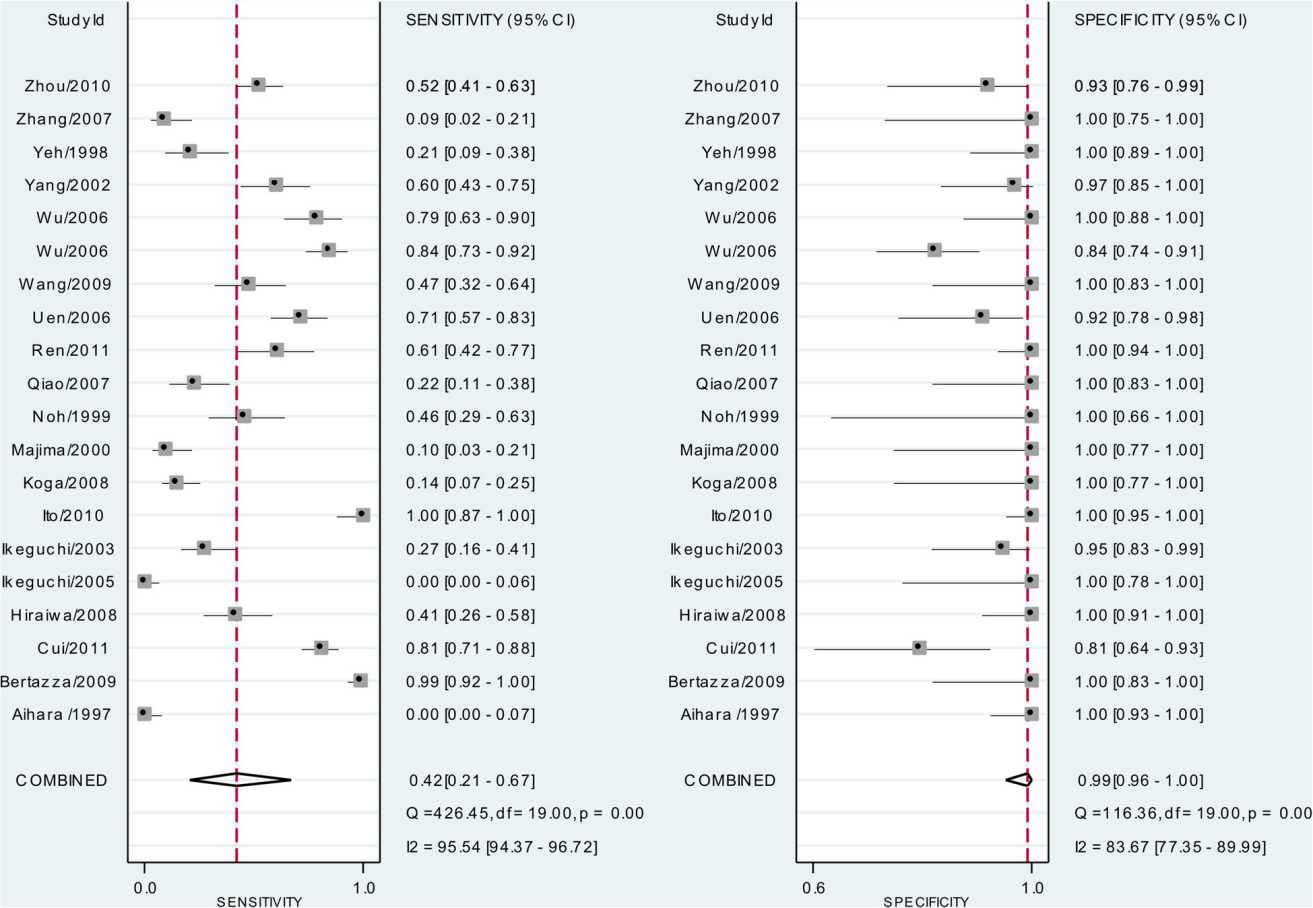
Forest plot showing study-specific (right-axis) and mean sensitivity and specificity with corresponding heterogeneity statistics.

**Figure 4:**
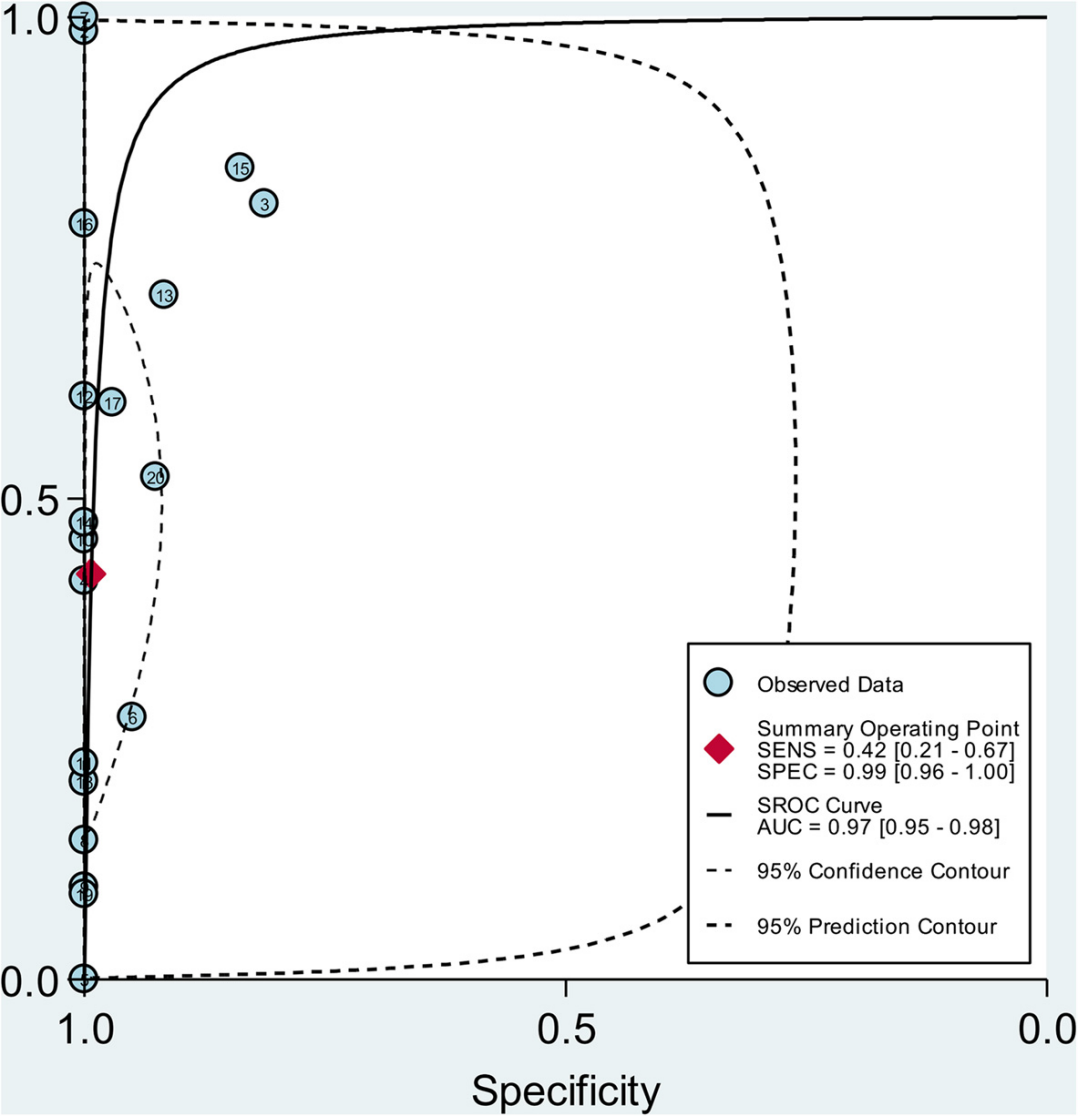
**Summary ROC curve with confidence and prediction regions around mean operating sensitivity and specificity point (The correspondence between numbers and the studies can be found in Additional file****1****: Table S2).**

The pooled SEN and SPE of CTC for the diagnosis of gastric cancer were 0.42 (95% confidence interval (CI), 0.21-0.67) and 0.99 (95% CI, 0.96-1.00) respectively (Figure 3, Table 2), with significant heterogeneity (*P* < 0.01, I^2^ = 95.54% and *P* < 0.01, I^2^ = 83.67%). Additionally, the pooled PLR was 58.2 (95% CI, 9.8-345.9) and the NLR was 0.58 (95% CI, 0.38-0.89) (Table 2). The DOR was 100 (95% CI, 15–663). Figure 4 presented the sROC curve for the included studies. The area under the curve (AUC) was 0.97 (95% CI 0.95–0.98).

**Table 2:** Pooled results of the meta-analysis of the diagnostic accuracy of CTCs detection in gastric cancer

**Analysis scenario**	**Sensitivity**	**Specificity**	**Positive LR**	**Negative LR**	**DOR**	Heterogeneity*
All studies	0.42 (0.21, 0.67)	0.99 (0.96, 1.00)	58.2 ( 9.8, 345.9)	0.58 (0.38, 0.89)	100 (15, 663)	98 (98, 99)
All studies without outliers	0.37 (0.16, 0.65)	0.99 (0.96, 1.00)	65.4 (8.4, 511.4)	0.63 (0.42, 0.96)	104 (11, 956)	94 (89, 99)
Subgroup: CEA	0.31 (0.10, 0.64)	0.94 (0.87, 0.98)	5.4 (2.1, 14.0)	0.73 (0.49, 1.09)	7 ( 2, 26)	98 (96, 99)
Subgroup: CK-19	0.27 (0.06, 0.67)	0.95 (0.90, 0.98)	5.4 (1.7, 16.4)	0.77 (0.50, 1.19)	7 (2, 31)	97 (96, 99)
Subgroup: CK-20	0.25 (0.13, 0.43)	0.95 (0.89, 0.98)	4.9 (1.6, 14.9)	0.79 (0.64, 0.98)	6 (2, 23)	0 (0, 100)
Subgroup: stage 1	0.22 (0.06, 0.56)	0.95 (0.89, 0.98)	4.3 (1.1, 17.7)	0.82 (0.59, 1.15)	5 (1, 29)	91 (83, 100)
Subgroup: stage 2	0.40 (0.14, 0.73)	0.96 (0.90, 0.98)	9.7 (4.5, 20.9)	0.62 (0.37, 1.07)	15 (5, 48)	93 (86, 99)
Subgroup: stage 3	0.46 (0.16, 0.80)	0.95 (0.90, 0.98)	9.4 (3.4, 25.9)	0.56 (0.28, 1.15)	17 (3, 83)	94 (89, 99)
Subgroup: stage 4	0.63 (0.43, 0.79)	0.97 (0.95, 0.98)	20.6 (11.2, 38.0)	0.38 (0.23, 0.64)	54 (21, 138)	71 (35,100)
Subgroup: stage 1-3	0.30 (0.09, 0.64)	0.96 (0.91, 0.98)	6.9 (2.2,21.3)	0.73 (0.48, 1.12)	9 (2, 42)	97 (95, 99)
Subgroup: PCR-based assay	0.39 (0.20, 0.60)	0.94 (0.90, 0.96)	6.1 (3.6, 10.4)	0.94 (0.90, 0.96)	9 (4, 21)	96 (95, 97)
Subgroup: immunological assay	0.82 (0.43, 1.00)	1.00 (0.98, 1.00)	74.5 (15.0,368.9)	0.335 (0.12-0.97)	340.9 (23.26,4996.7)	93 (88, 97)

Numbers in parentheses are 95% CIs. *DOR* diagnostic odds ratio, *LR* likelihood ratio.

* Inconsistency indexes are percentages.

**Figure 5:**
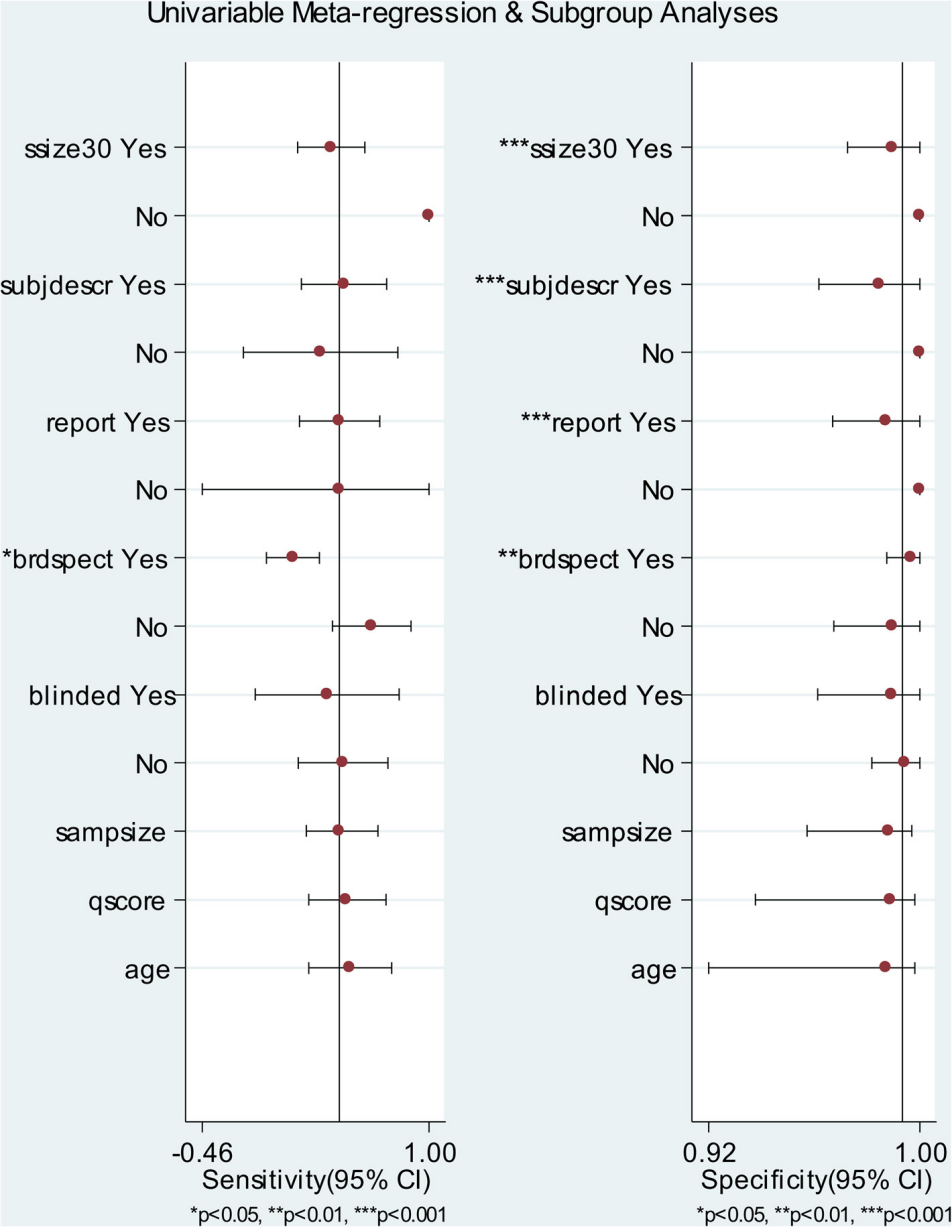
Forest plot of multiple univariable meta-regression and subgroup analyses for SEN and SPE.

The proportion of heterogeneity likely due to threshold effect was 19%, which meant a slight influence of a diagnostic threshold effect. To explore other potential heterogeneities, meta-regression and subgroup meta-analysis were performed (Figure 5). Overall, the test performances varied by patient population, study design and study quality. The pooled SPE was lower with some covariates, such as study size greater than 30 (*P* < 0.001), adequate description of study subjects (*P* < 0.001), satisfactory reporting of results (*P* < 0.001) and broad spectrum of disease (*P* < 0.01).

**Figure 6:**
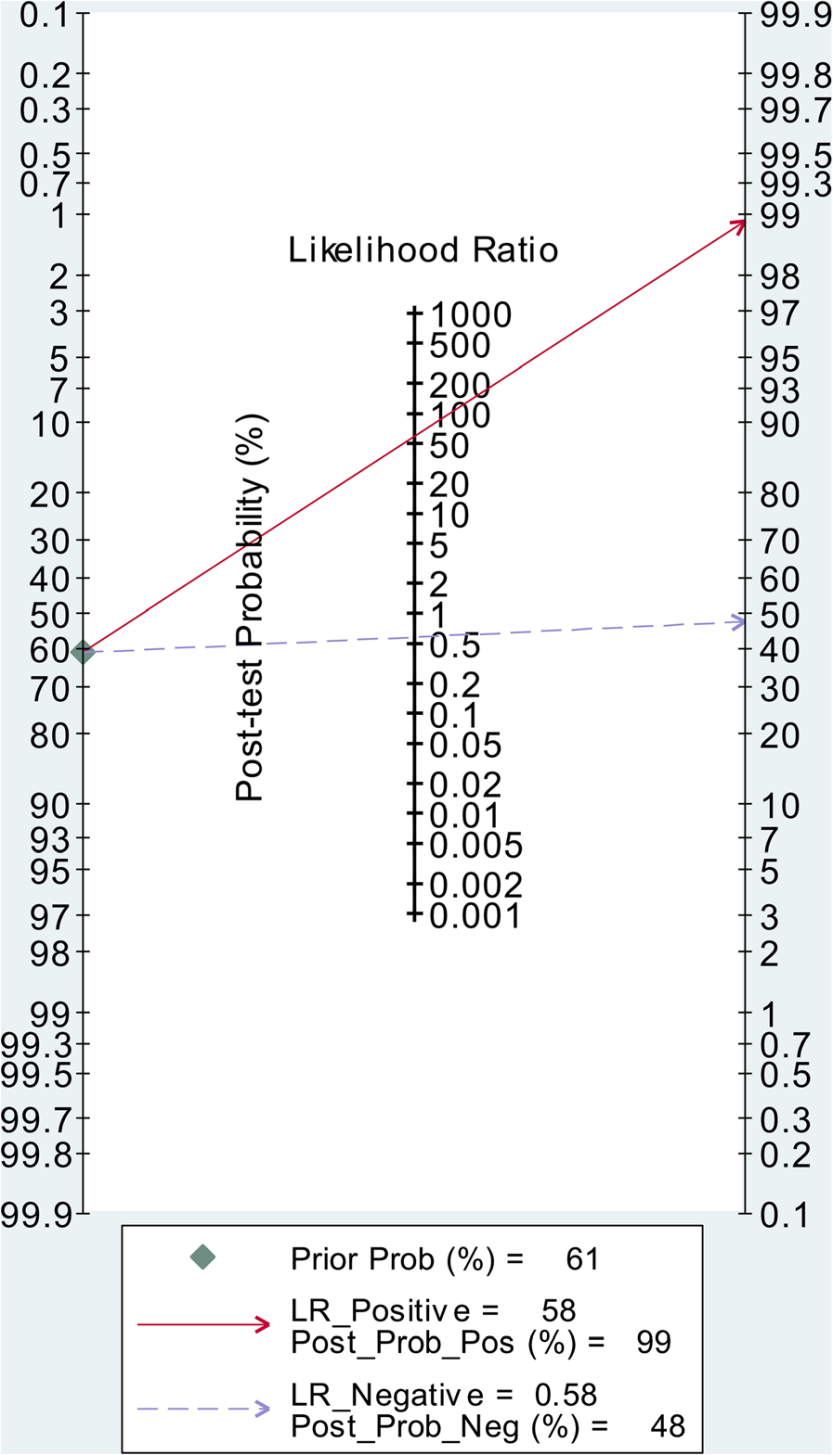
Fagan plot analysis to evaluate the clinical utility of CTCs detection.

As shown in the Fagan plot (Figure 6), with a pre-test probability of gastric cancer of 61% in this meta-analysis, the posttest probability of gastric cancer, given a negative CTCs detection result, was 48%, while 99% with a positive result.

**Figure 7:**
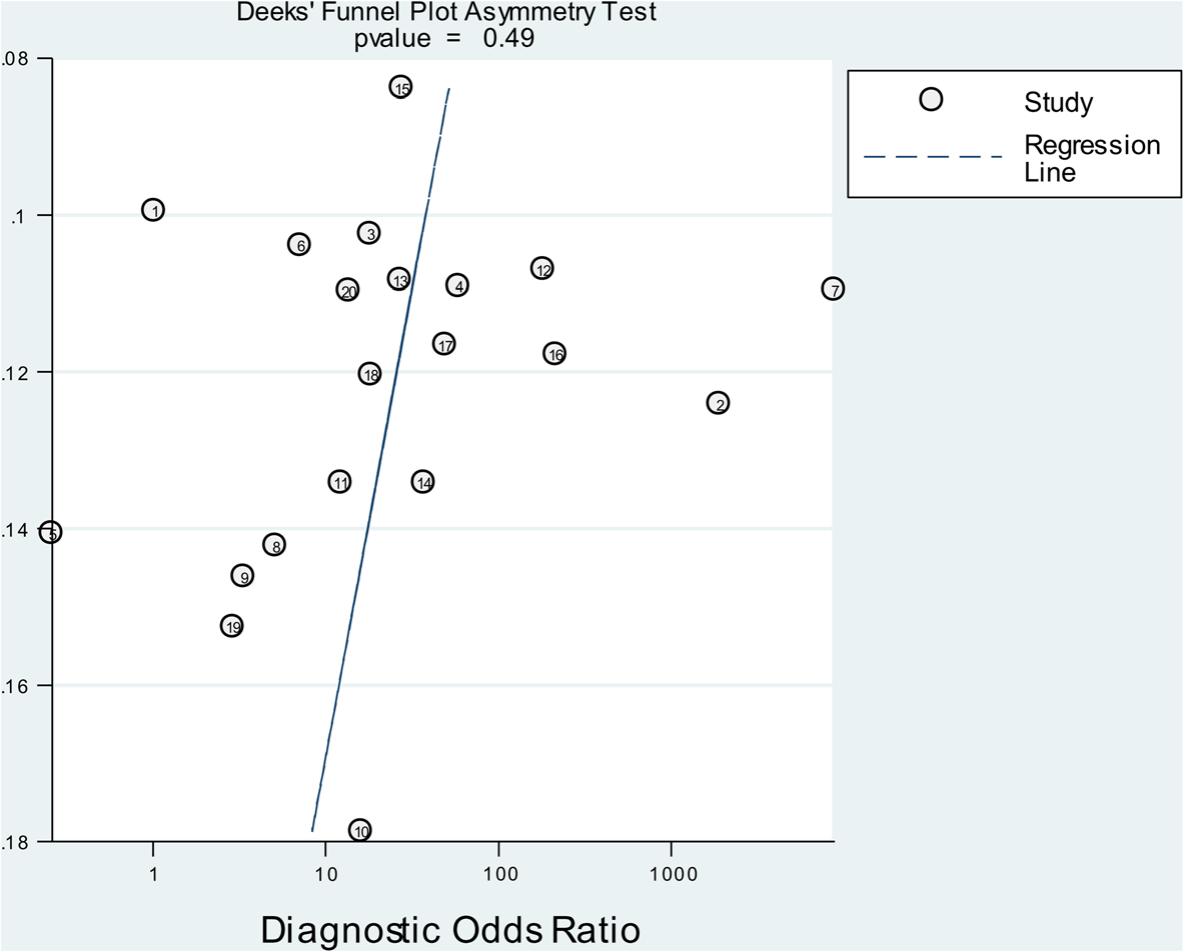
Funnel plot with superimposed regression line.

**Figure 8:**
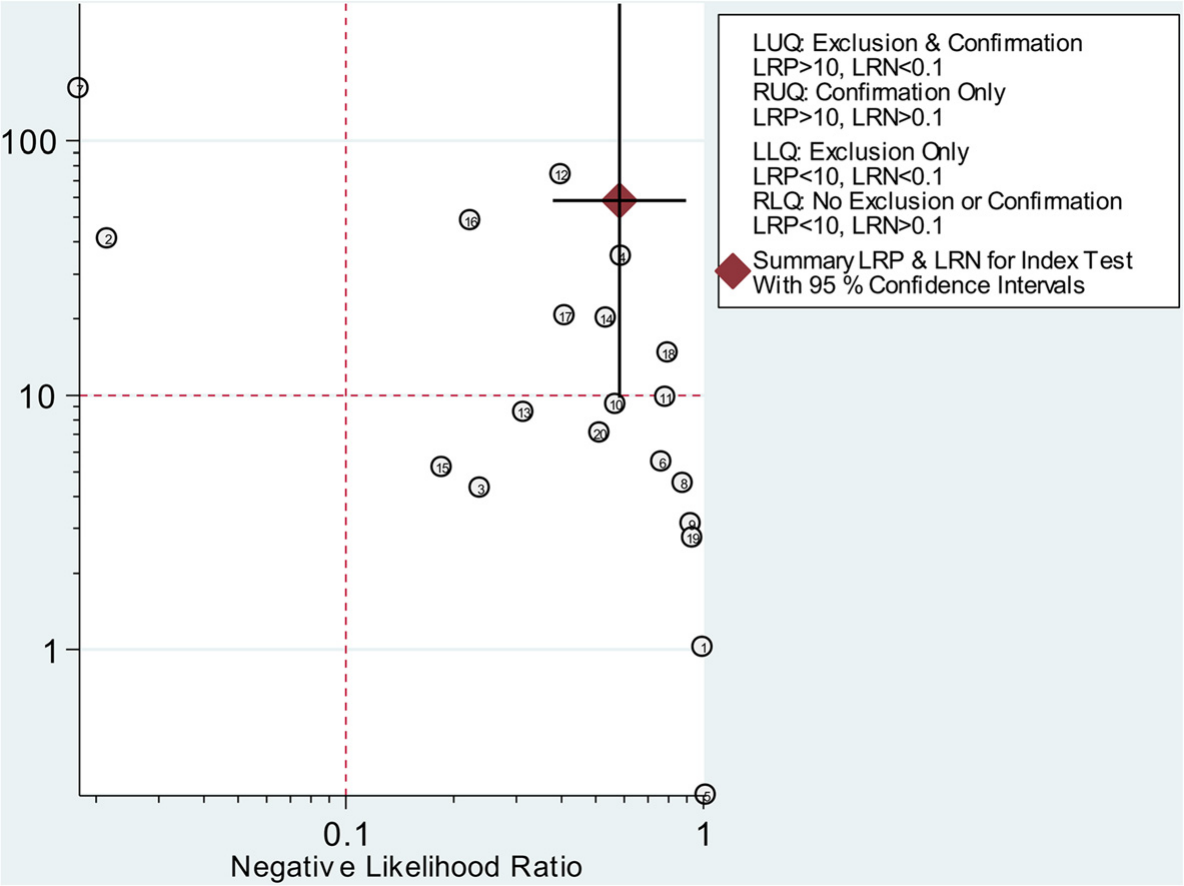
Likelihood ratio scattergram.

According to the Deek’s funnel plot asymmetry test, the *P* value was 0.49 for the slope coefficient, which showed there was not a significant publication bias (Figure 7). The likelihood ratio scattergram (Figure 8) showing summary point of likelihood ratios obtained as functions of mean SEN and specificity in the right upper quadrant suggested that the CTCs detection was useful for the confirmation of presence of gastric cancer (when positive) but not for its exclusion (when negative) [23]. The predictive values and probability modifying plot was shown in Additional file 1: Figure S2.

The pooled SEN, SPE, PLR, NLR, DOR and the AUC mentioned above were summarized in Table 2.

### Diagnostic accuracy of CTCs detection in different markers (subgroup analysis)

**Figure 9:**
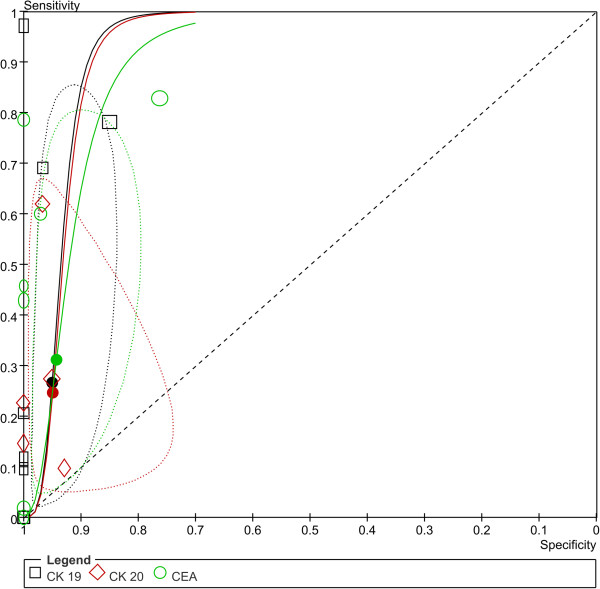
**Summary ROC plot of SEN and SPE of CK 19, Ck 20, and CEA based CTCs detections.** (Dotted ellipses around the spots represent the 95% CI around the summary estimates. The diamonds, rectangles and circles represent individual studies and size of the diamonds/rectangles/circles is proportional to the number of patients included in the study).

8 studies reported data about CK-19 [19,21,22,33,36,37,43,45], 5 about CK-20 [19,33,36,37,39], and 8 about CEA [19,22,33,34,38,43,44,46]. There were no significant differences between the three biomarkers (Figure 9, Additional file 1: Figure S3).

### Diagnostic accuracy of CTCs detection in different phases (subgroup analysis)

**Figure 10:**
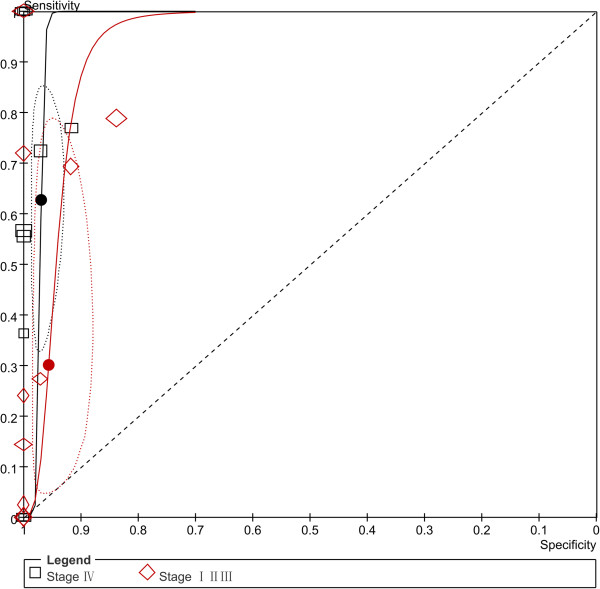
**Summary ROC plot of SEN and SPE of CTCs detection in stage I to III, and IV gastric cancer patients.** (Dotted ellipses around the spots represent the 95% CI around the summary estimates. The diamonds and rectangles and circles represent individual studies and size of the diamonds/rectangles is proportional to the number of patients included in the study).

10 studies [19-21,34,35,37,38,41,43,44] reported data about patients with stage I to III gastric cancer, and stage IV. Figure 10 and Additional file 1: Figure S4 showed that the SEN of CTCs detection in stage IV patients was higher than in stage I to III, more specifically, the SEN was higher in more advanced stage than earlier stage (Additional file 1: Figure S5 and S6) while the SPE was almost on the same level.

### Diagnostic accuracy of CTCs detection in different detection methods (subgroup analysis)

There are two main methods for CTCs detection which are PCR-based assays both exploiting tissue and/or tumor specific antigens and immunological assays using monoclonal antibodies [48]. In this meta-analysis, the included studies can also be divided into two major groups. One is the PCR-based assay group [19,21,22,32-34,36-39,41-47] while the other is immunological assay [20,35,40]. The pooled sensitivity of two group were 0.35 (95% CI, 0.11-0.59), and 0.82 (95% CI, 0.43-1.00) respectively. And the heterogeneity were P < 0.01, I^2^ = 95.9% and P < 0.01, I^2^ = 80.0%.

### Sensitivity analysis

**Figure 11:**
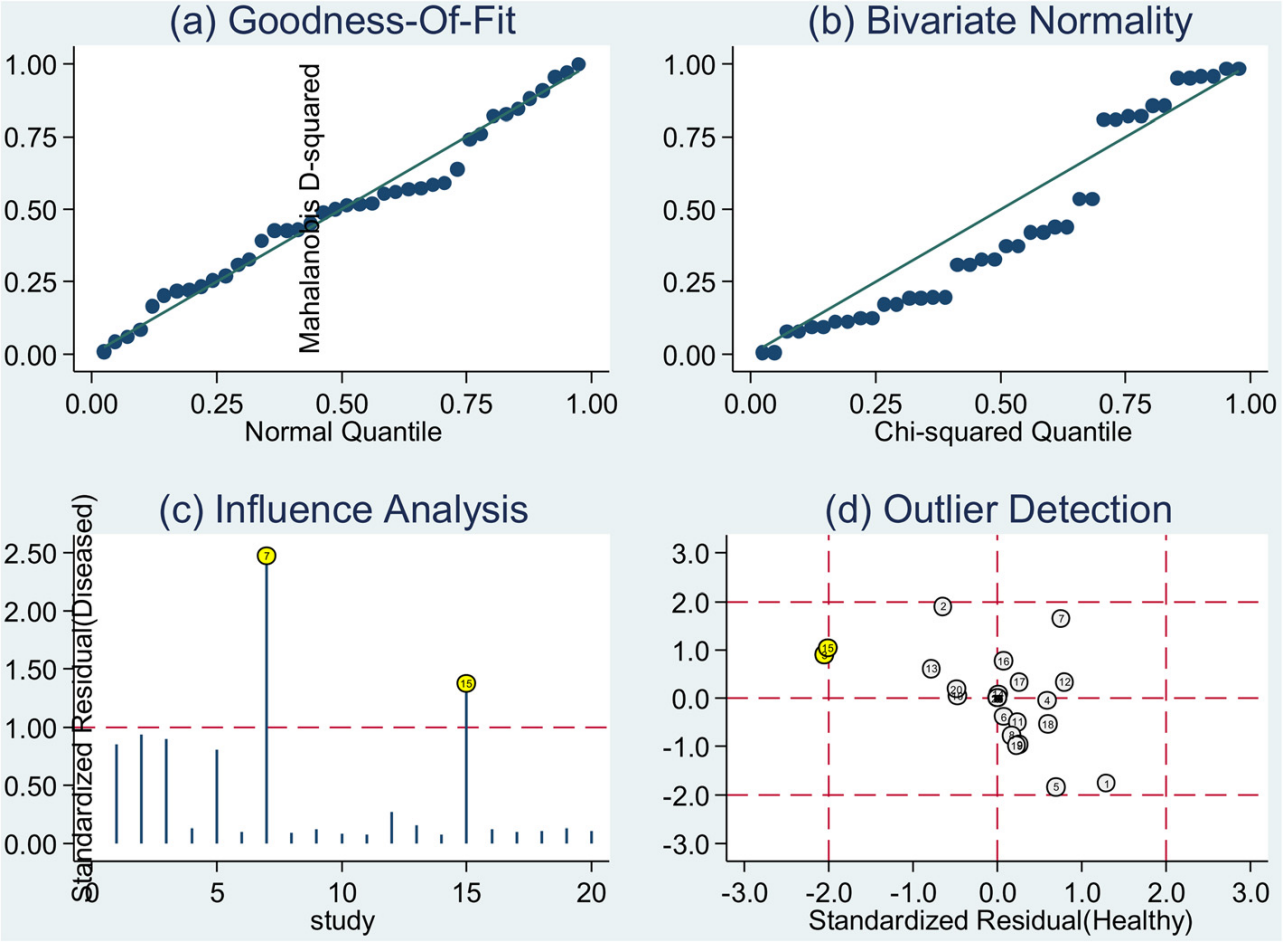
Graphical depiction of residual-based goodness-of-fit (A), bivariate normality (B), influence and outlier detection analyses (C and D, respectively).

Figure 11d showed two outlier studies [32,43]. After the exclusion of these two studies, the I^2^ for heterogeneity decreased from 99% to 94%, the SEN decreased from 0.42 to 0.37, PLR increased from 58.2 to 65.4, NLR increased from 0.58 to 0.63, and DOR increased from 100 to 104, while SPE had minimal change (Table 2).

## Discussion

Recently, the detection of circulating cancer cells in peripheral blood has received growing enthusiasm in the diagnosis of various cancers. However, the diagnostic accuracy varied in different studies. There were several meta-analyses about CTCs detection in cancers. In Tsao’s meta-analysis [49], tyrosinase messenger RNA was positive in 18% patients with stage I cutaneous melanoma disease, 28% with stage II disease, 30% with stage III disease, and 45% with stage IV disease. Specificities were 1.00 in all but 1 study. A meta-analysis conducted by Zhang *et al.*[50] showed SEN and SPE of CTCs detection in patients with lung cancer were 0.80 and 0.77, respectively. Msaouel and Koutsilieris *et al.*[11] reported that the overall SEN and SPE of CTCs detection in patients with bladder and urothelial cancer were 0.351 and 0.894, respectively. This current study is the first meta-analysis focusing on the diagnostic value of CTCs detection in peripheral blood of gastric cancer patients.

In this meta-analysis, CTCs detection in peripheral blood of patients with gastric cancer had limited diagnostic value, because it failed to identify more than half of the patients (SEN is only 0.42). Compared with the meta-analyses mentioned above [11,50], the SEN in gastric cancer was higher than that in bladder and urothelial caner, while lower than lung cancer. However, the SPE was high (0.99). These indicated that CTCs detection might not be qualified as screening test, but useful in the confirmation of gastric cancer. The SPE in gastric cancer was almost the same as in lung cancer, while higher than that in bladder and urothelial cancer. Thus, it can be concluded that the confirmative value of CTCs detection in gastric cancer was lower than that in lung cancer, but higher than that in bladder and urothelial cancer. The pooled PLR was 58.2, which indicated that CTCs detection can confirm this disease, because few patients would be falsely diagnosed as gastric cancer with positive CTCs detection, whereas, patients might still have gastric cancer even though the results are negative because the NLR was only 0.58, which meant CTCs detection couldn’t rule out the disease by the negative results. It should be noted that the high DOR (100) as well as the high AUC (0.97) reflecting an overall high diagnostic accuracy by CTCs detection. According to the likelihood ratio scattergram, the plot showed that CTCs detection could be useful for the confirmation of presence of gastric cancer (when positive) but not for its exclusion (when negative).

There are various kinds of PCR based markers used in the detection of CTCs, and they can be divided into two categories. One is expressed by almost all the tumor cells originated form epithelial cells, such as epithelial markers (cytokeratins (CK), epithelial cell adhesion molecule (EpCAM), human epithelial antigen (HEA)). The other is tumor cell-specific markers that are expressed by a particular type of cancer, such as CEA, a-Foetoprotein, Her2-neu, CA-IX and prostate specific antigen (PSA) [17,51]. However, only 3 markers were investigated in more than three studies in this meta-analysis, so subgroup analyses were performed targeting these 3 markers. The results showed that these three markers had similar SEN and SPE, and showed less significant advantage than pooled SEN and SPE. On the other hand, we found that the diagnostic SEN of CTCs detection was higher in more advanced tumor stage. CTCs were released from the primary tumor or metastasis, so it was reasonable to detect them in stage IV patients more easily. It was reported that the CTCs detection in malignant melanoma had correlated with clinical stage and had been an independent prognostic factor for the disease recurrence [52,53]. Identifying small amounts of tumor cells by CTCs detection could prove the presence of micrometastasis in peripheral blood, but hardly by other technologies such as pathology and radiology. Thus, for patients who had positive CTCs detection results, postoperative adjuvant chemotherapy or radiotherapy was highly recommended. This association indicated that CTCs detection might be helpful in therapy of gastric cancer, especially for those who were more likely to have advanced cancer.

An important consideration in this meta-analysis was its limitations. First of all, as in other diagnostic test accuracy reviews, the basic characteristics of included studies were not coherent. The time of sample collection was not consistent. If the samples were collected after surgeries, the circulating cancer cells might be released into the peripheral blood due to surgeries, which would increase the SEN, whereas, if the samples were collected after the chemotherapy, the CTCs in the peripheral blood might be killed. Moreover, 12 studies didn’t collect two consecutive blood samples to avoid contamination by epithelial cells. And CTCs detection diagnostic accuracy might be higher in studies in which larger blood volumes were collected. A conference abstract [35] was also included, in which the basic characteristic was unclear and the scores of QUADAS and STARD couldn’t be obtained without full text. What’s more, according to the meta-regression, the sample size less than 30 introduced significant heterogeneity (*P* < 0.001). In addition, as we known, the ideal method to detect CTCs should focus on the tumor cells directly, for example cytopathology, not the surrogate markers indirectly linked to tumor cells as studied in the included papers. However, the concentration of CTCs in blood stream is low, as a result, the isolation and detection of CTCs is not an easy process [54]. So PCR-based assay both exploiting tissue and/or tumor specific antigens and immunological assay using monoclonal antibodies were developed to detect CTCs indirectly. Different methods may increase the heterogeneity in meta-analysis, so subgroup analysis was conducted based on the method. The pooled SEN of the two group had no statistically significant difference (*P* = 0.10), and the heterogeneity still existed in both group. What’s more, we performed a subgroup analysis according to the published years. We divided the PCR-based assay group into three groups, which were 1997–2002 group, 2003–2007 group and 2008–2012 group. The pooled SEN of three subgroups were 0.17 (95% CI, 0.04-0.52), 0.31 (95% CI, 0.08-0.71) and 0.67 (95% CI, 0.27-0.92), respectively (Additional file 2: Figure S7, Additional file 3: Figure S8). The SEN has a trend of increase with the development of times. So we believe that with the development detection technology, we may get an ideal conclusion when updating this meta-analysis in the future.

Apart from all the items mentioned above might contribute to the significant inter-study heterogeneity, the outlier studies could also introduce heterogeneity [55]. According to Figure 11, there were 2 outliers [32,43] in this meta-analysis, after the exclusion of the two outliers, the heterogeneity did not change much, which meant there were other potential factors resulting in the significant heterogeneity, for example, the differences in CTCs enrichment and identification techniques and biomarkers. In this meta-analysis, the diagnostic threshold effect and publication bias didn’t introduce significant heterogeneity. In order to explore other potential heterogeneities, meta-regression and subgroup meta-analysis were performed, and heterogeneity was found in sample size, description of study subjects, reporting of results and spectrum of the diseases in control group. Therefore, multi-center studies with standardized study designs were needed to decrease inter-study heterogeneity.

To include all the eligible studies as many as possible and diminish the language bias in this systematic review, we didn’t apply any restrictions about the language when we searched the database, such as PubMed, Embase. Meanwhile we used Wanfang Database as a supplementary database to collect the non-English language publications. Despite this, there should be some other language publications which are not included in our systematic review, such as German or Japanese literatures. According to the included studies in this systematic review, it is easy found that nearly all of the patients and controls were Asians, so the clinical significance may have its limitation. More studies about Caucasians are needed to explore the diagnostic value of CTCs detection.

Finally, although we search for studies without the limitation of time and languages, we didn’t search for unpublished data. Diagnostic studies are easy to undertake and are not usually recorded on research registries, so it is difficult for researchers to search for unpublished data. Therefore, some missing and unpublished data may not be included in current study, which may overestimate the pooled results.

## Conclusions

In summary, with lower and inconsistent SEN estimates for CTCs detection in GC, CTCs detection alone cannot be recommended as a screening test of GC. However, it might be used as a noninvasive method for the confirmation of the gastric cancer diagnosis because of the high SPE.

## Competing interests

The authors declare that they have no competing interests.

## Authors’ contributions

MZ designed this systematic review. LT and SZ have been involved in the search strategy. LT, WL, JH and YT did the collection and the analysis of the data. LT, SZ and PG interpreted the data. LT wrote the systematic review and all the other authors revised the manuscript. NFP provided general advice on the manuscript. All the authors read and approved the final manuscript.

## Pre-publication history

The pre-publication history for this paper can be accessed here:

http://www.biomedcentral.com/1471-2407/13/314/prepub

## Supplementary Material

Additional file 1: Figure S1 Paired forest plot depiction of empirical Bayes predicted versus observed sensitivity and specificity. **Figure S2.** Probability Modifying Plot. **Figure S3.** Forest plots of sensitivity and specificity of CK 19, Ck 20, and CEA based CTCs detections. **Figure S4.** Forest plots of sensitivity and specificity of CTCs detection in stage I to III, and IV gastric cancer patients. **Figure S5.** Forest plots of sensitivity and specificity of CTCs detection in stage I, II, III, and IV gastric cancer patients. **Figure S6.** Summary ROC plot of SEN and SPE of CTCs detection in stage I, II, III, and IV gastric cancer patients. (Dotted ellipses around the spots represent the 95% CI around the summary estimates. The diamonds, rectangles and circles represent individual studies and size of the diamonds/rectangles/circles is proportional to the number of patients included in the study). **Table S1.** Main characteristics of studies included in the meta-analysis of the diagnostic accuracy of CTCs detection in gastric cancer. **Table S2.** The correspondence between numbers and the studies. Click here for file

Additional file 2: Figure S7 Forest plots of sensitivity and specificity of CTCs detection in different published years among PCR-based group. Click here for file

Additional file 3: Figure S8 Summary ROC plot of SEN and SPE of CTCs detection in different published years among PCR-based group. (Dotted ellipses around the spots represent the 95% CI around the summary estimates. The diamonds, rectangles and circles represent individual studies and size of the diamonds/rectangles/circles is proportional to the number of patients included in the study). Click here for file
