# Supplementary figures and images for: Diagnostic accuracy of circulating tumor cells detection in gastric cancer: systematic review and meta-analysis
Source: BMC Cancer. 2013 Jun 27;13:314. doi: 10.1186/1471-2407-13-314 (PMC3699416; doi:10.1186/1471-2407-13-314)

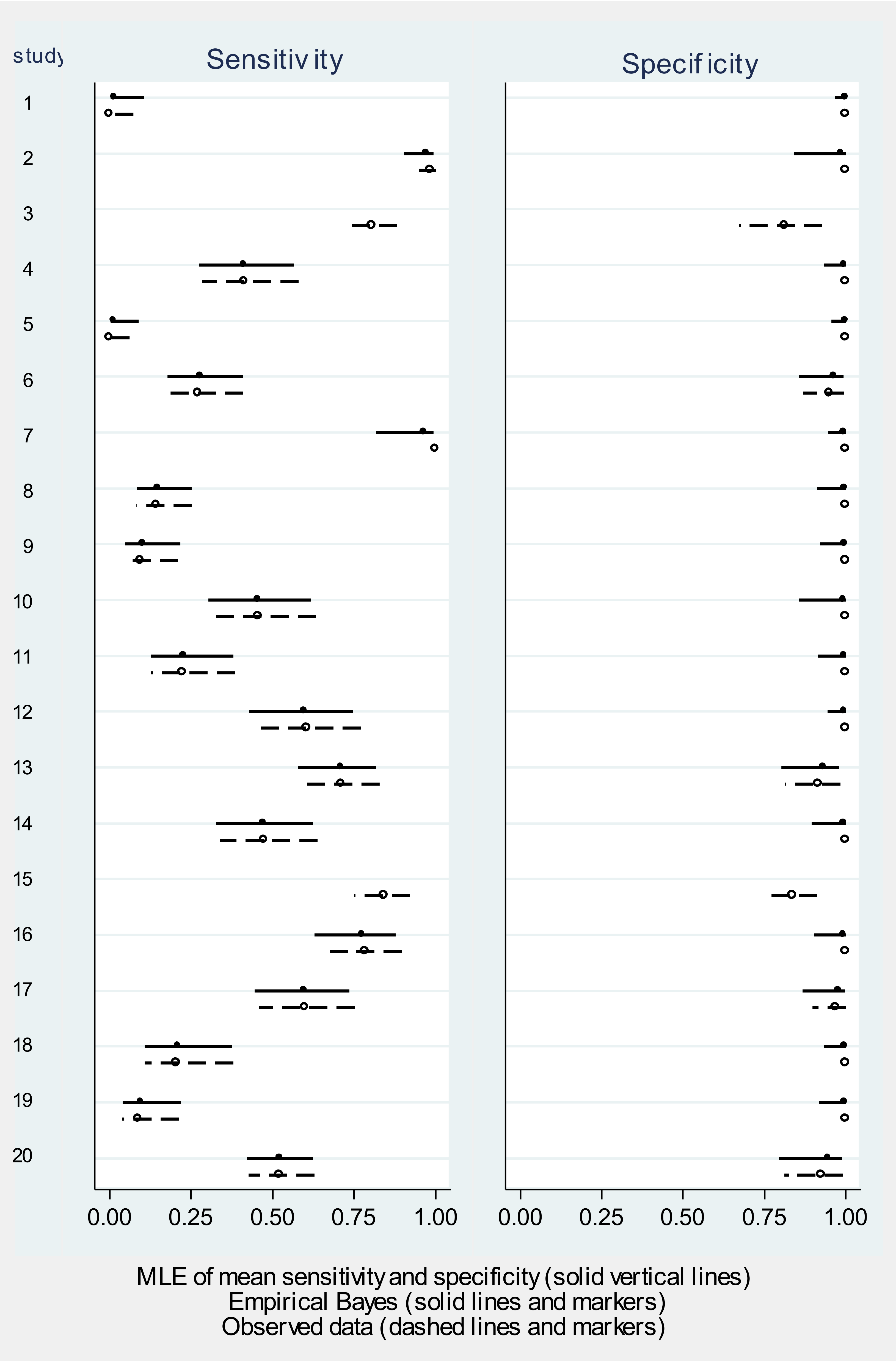

Supplement: Additional file 1: Figure S1 — Paired forest plot depiction of empirical Bayes predicted versus observed sensitivity and specificity. Figure S2. Probability Modifying Plot. Figure S3. Forest plots of sensitivity and specificity of CK 19, Ck 20, and CEA based CTCs detections. Figure S4. Forest plots of sensitivity and specificity of CTCs detection in stage I to III, and IV gastric cancer patients. Figure S5. Forest plots of sensitivity and specificity of CTCs detection in stage I, II, III, and IV gastric cancer patients. Figure S6. Summary ROC plot of SEN and SPE of CTCs detection in stage I, II, III, and IV gastric cancer patients. (Dotted ellipses around the spots represent the 95% CI around the summary estimates. The diamonds, rectangles and circles represent individual studies and size of the diamonds/rectangles/circles is proportional to the number of patients included in the study). Table S1. Main characteristics of studies included in the meta-analysis of the diagnostic accuracy of CTCs detection in gastric cancer. Table S2. The correspondence between numbers and the studies. [file 1471-2407-13-314-S1.zip › Supplementary Figure 1.tif]

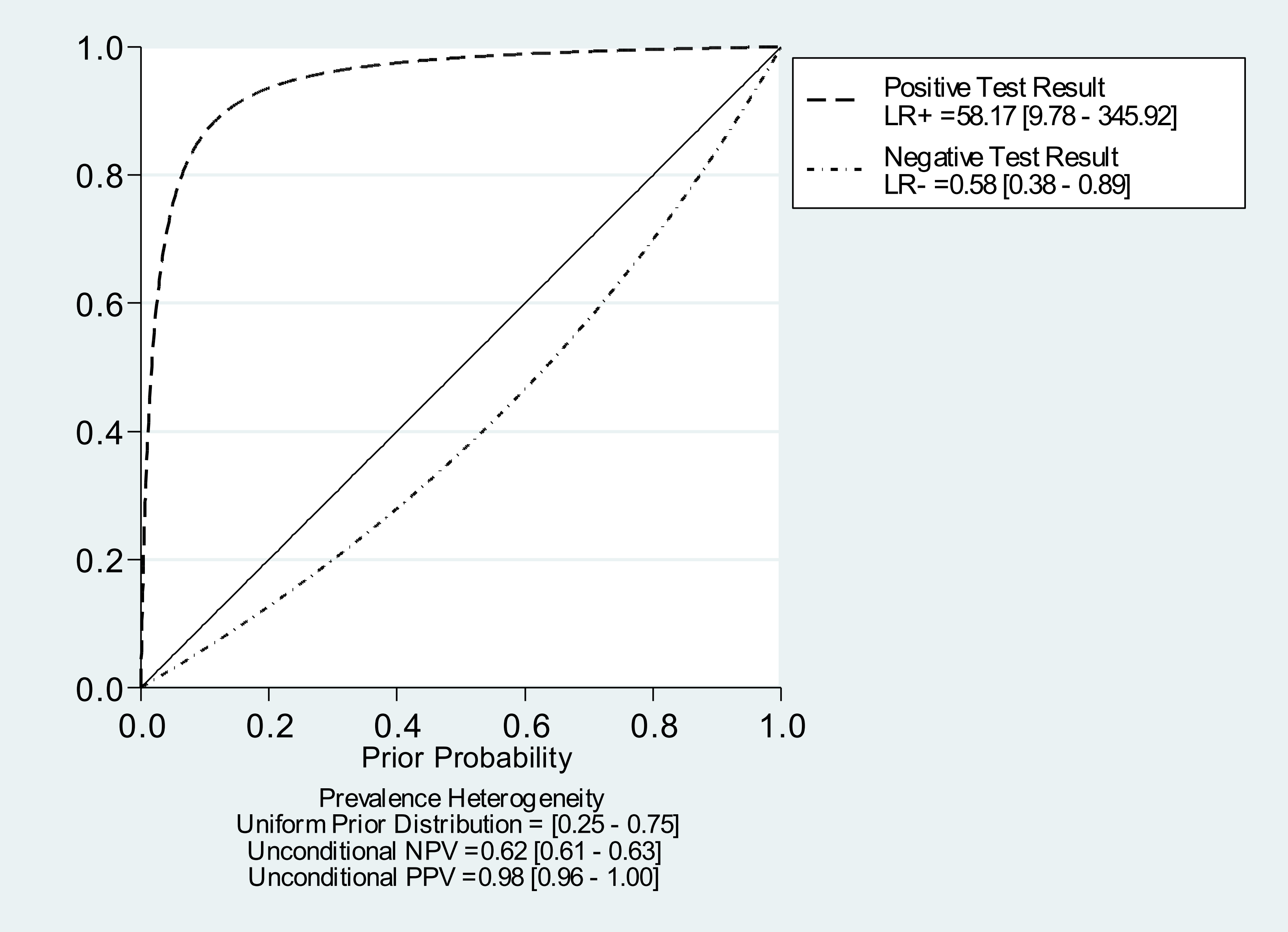

Supplement: Additional file 1: Figure S1 — Paired forest plot depiction of empirical Bayes predicted versus observed sensitivity and specificity. Figure S2. Probability Modifying Plot. Figure S3. Forest plots of sensitivity and specificity of CK 19, Ck 20, and CEA based CTCs detections. Figure S4. Forest plots of sensitivity and specificity of CTCs detection in stage I to III, and IV gastric cancer patients. Figure S5. Forest plots of sensitivity and specificity of CTCs detection in stage I, II, III, and IV gastric cancer patients. Figure S6. Summary ROC plot of SEN and SPE of CTCs detection in stage I, II, III, and IV gastric cancer patients. (Dotted ellipses around the spots represent the 95% CI around the summary estimates. The diamonds, rectangles and circles represent individual studies and size of the diamonds/rectangles/circles is proportional to the number of patients included in the study). Table S1. Main characteristics of studies included in the meta-analysis of the diagnostic accuracy of CTCs detection in gastric cancer. Table S2. The correspondence between numbers and the studies. [file 1471-2407-13-314-S1.zip › Supplementary Figure 2.tif]

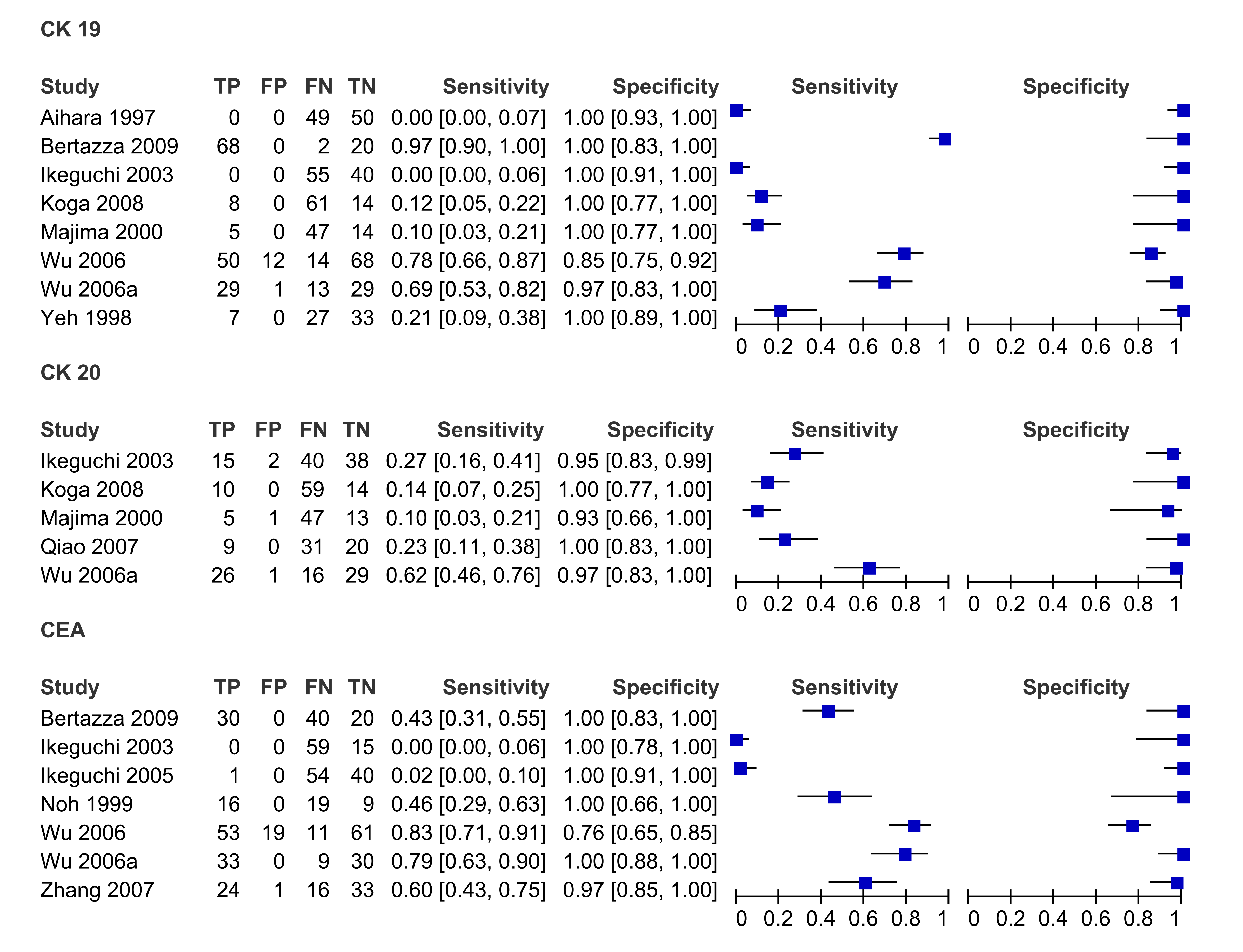

Supplement: Additional file 1: Figure S1 — Paired forest plot depiction of empirical Bayes predicted versus observed sensitivity and specificity. Figure S2. Probability Modifying Plot. Figure S3. Forest plots of sensitivity and specificity of CK 19, Ck 20, and CEA based CTCs detections. Figure S4. Forest plots of sensitivity and specificity of CTCs detection in stage I to III, and IV gastric cancer patients. Figure S5. Forest plots of sensitivity and specificity of CTCs detection in stage I, II, III, and IV gastric cancer patients. Figure S6. Summary ROC plot of SEN and SPE of CTCs detection in stage I, II, III, and IV gastric cancer patients. (Dotted ellipses around the spots represent the 95% CI around the summary estimates. The diamonds, rectangles and circles represent individual studies and size of the diamonds/rectangles/circles is proportional to the number of patients included in the study). Table S1. Main characteristics of studies included in the meta-analysis of the diagnostic accuracy of CTCs detection in gastric cancer. Table S2. The correspondence between numbers and the studies. [file 1471-2407-13-314-S1.zip › Supplementary Figure 3.tif]

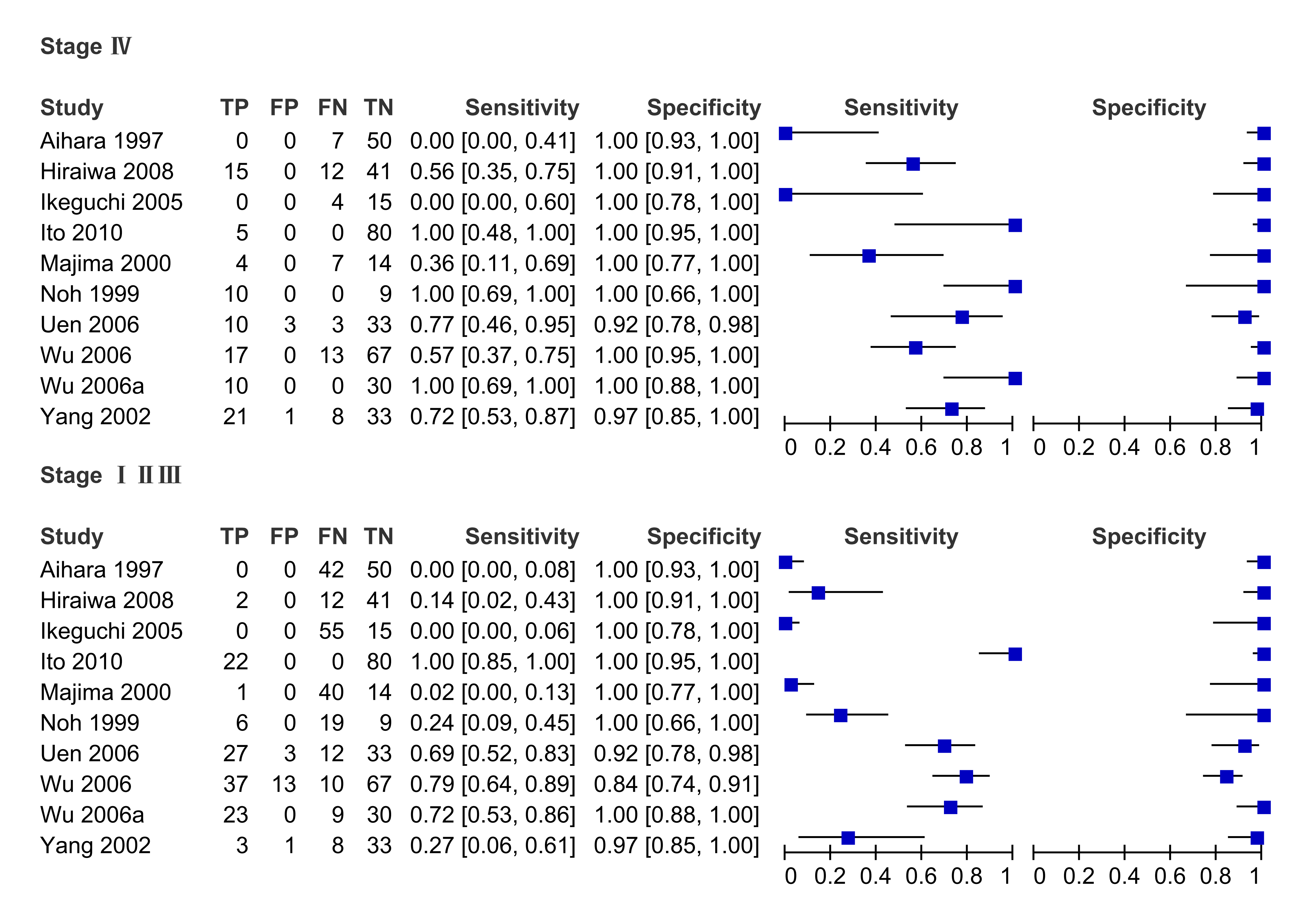

Supplement: Additional file 1: Figure S1 — Paired forest plot depiction of empirical Bayes predicted versus observed sensitivity and specificity. Figure S2. Probability Modifying Plot. Figure S3. Forest plots of sensitivity and specificity of CK 19, Ck 20, and CEA based CTCs detections. Figure S4. Forest plots of sensitivity and specificity of CTCs detection in stage I to III, and IV gastric cancer patients. Figure S5. Forest plots of sensitivity and specificity of CTCs detection in stage I, II, III, and IV gastric cancer patients. Figure S6. Summary ROC plot of SEN and SPE of CTCs detection in stage I, II, III, and IV gastric cancer patients. (Dotted ellipses around the spots represent the 95% CI around the summary estimates. The diamonds, rectangles and circles represent individual studies and size of the diamonds/rectangles/circles is proportional to the number of patients included in the study). Table S1. Main characteristics of studies included in the meta-analysis of the diagnostic accuracy of CTCs detection in gastric cancer. Table S2. The correspondence between numbers and the studies. [file 1471-2407-13-314-S1.zip › Supplementary Figure 4.tif]

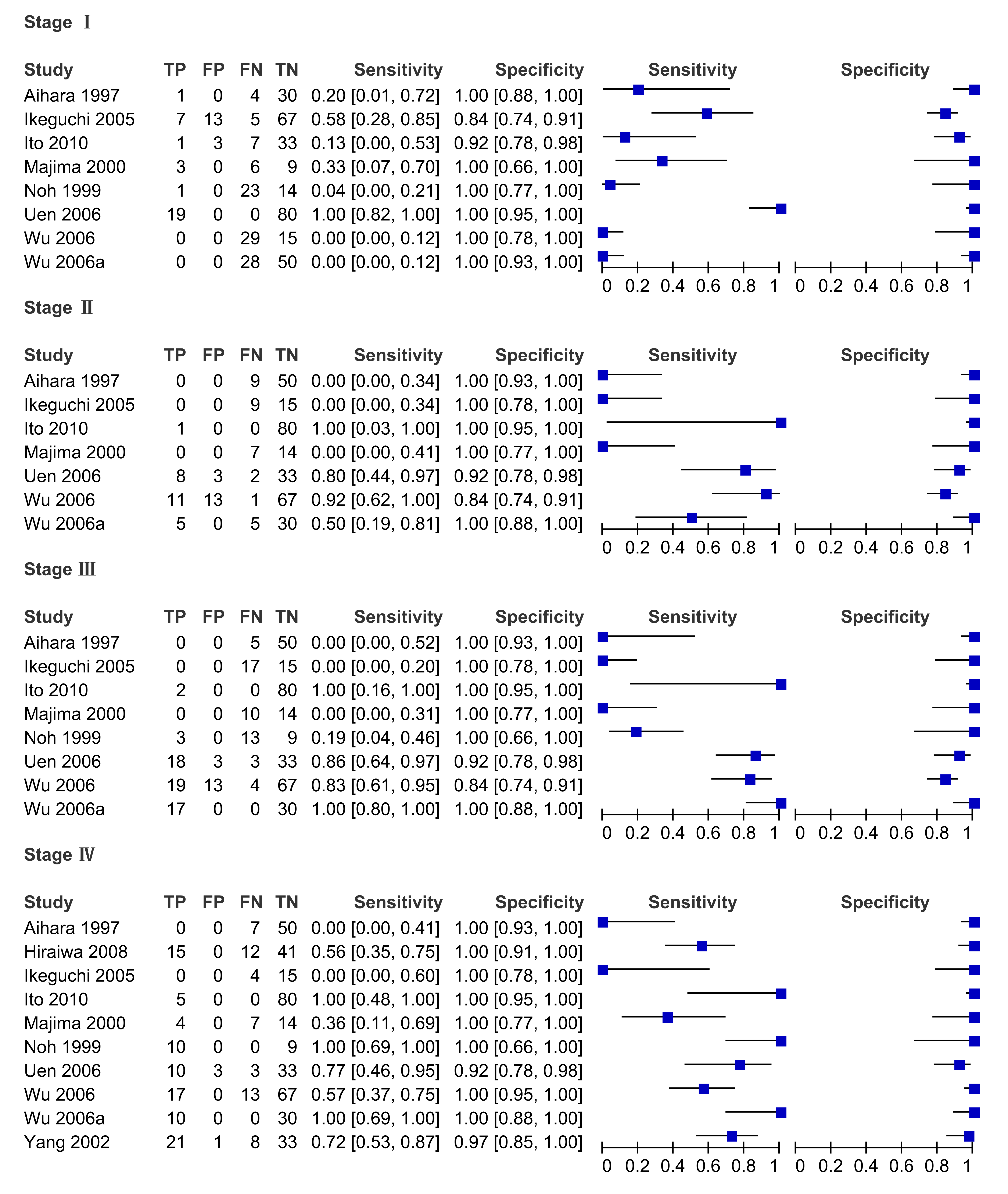

Supplement: Additional file 1: Figure S1 — Paired forest plot depiction of empirical Bayes predicted versus observed sensitivity and specificity. Figure S2. Probability Modifying Plot. Figure S3. Forest plots of sensitivity and specificity of CK 19, Ck 20, and CEA based CTCs detections. Figure S4. Forest plots of sensitivity and specificity of CTCs detection in stage I to III, and IV gastric cancer patients. Figure S5. Forest plots of sensitivity and specificity of CTCs detection in stage I, II, III, and IV gastric cancer patients. Figure S6. Summary ROC plot of SEN and SPE of CTCs detection in stage I, II, III, and IV gastric cancer patients. (Dotted ellipses around the spots represent the 95% CI around the summary estimates. The diamonds, rectangles and circles represent individual studies and size of the diamonds/rectangles/circles is proportional to the number of patients included in the study). Table S1. Main characteristics of studies included in the meta-analysis of the diagnostic accuracy of CTCs detection in gastric cancer. Table S2. The correspondence between numbers and the studies. [file 1471-2407-13-314-S1.zip › Supplementary Figure 5.tif]

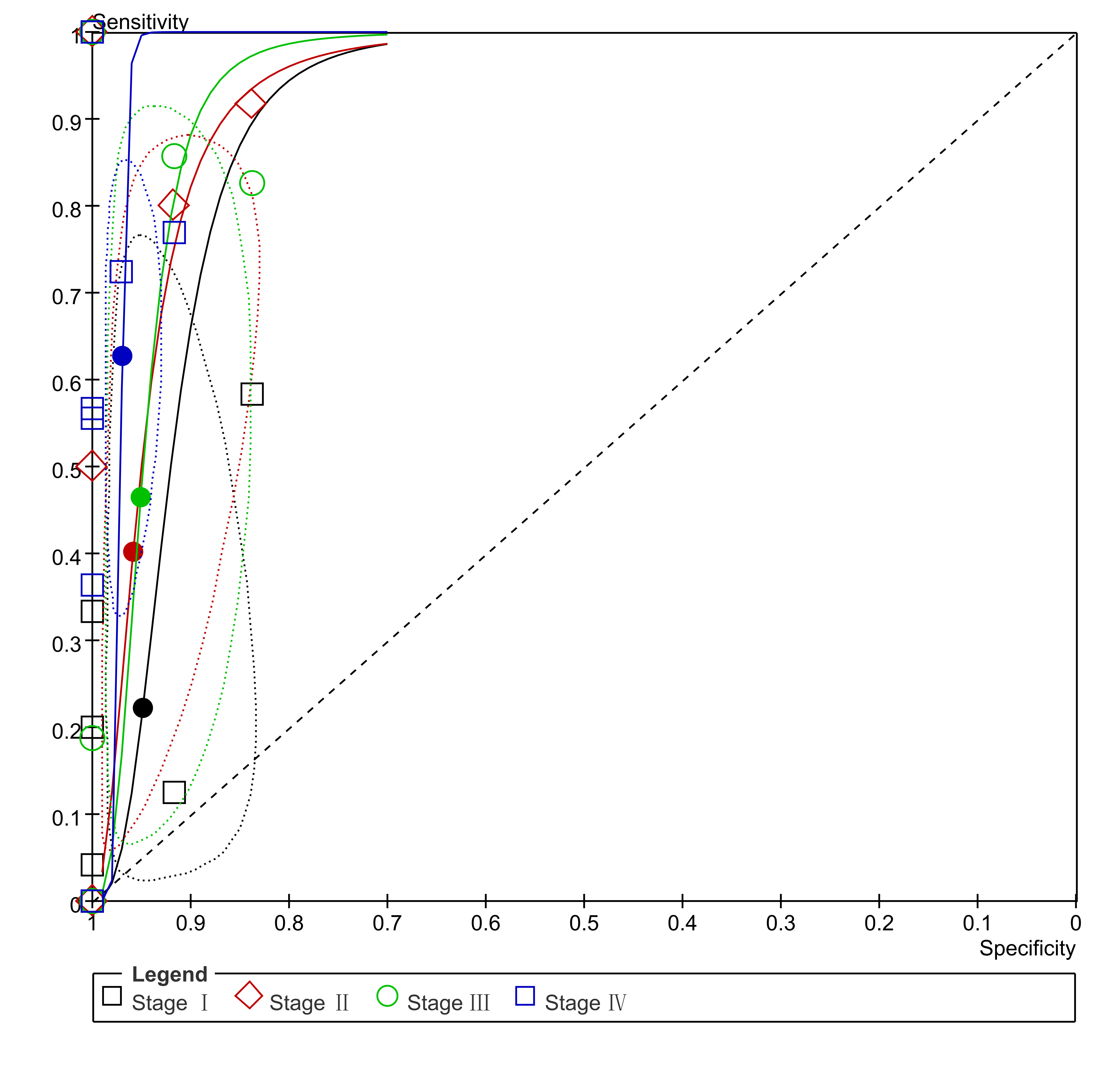

Supplement: Additional file 1: Figure S1 — Paired forest plot depiction of empirical Bayes predicted versus observed sensitivity and specificity. Figure S2. Probability Modifying Plot. Figure S3. Forest plots of sensitivity and specificity of CK 19, Ck 20, and CEA based CTCs detections. Figure S4. Forest plots of sensitivity and specificity of CTCs detection in stage I to III, and IV gastric cancer patients. Figure S5. Forest plots of sensitivity and specificity of CTCs detection in stage I, II, III, and IV gastric cancer patients. Figure S6. Summary ROC plot of SEN and SPE of CTCs detection in stage I, II, III, and IV gastric cancer patients. (Dotted ellipses around the spots represent the 95% CI around the summary estimates. The diamonds, rectangles and circles represent individual studies and size of the diamonds/rectangles/circles is proportional to the number of patients included in the study). Table S1. Main characteristics of studies included in the meta-analysis of the diagnostic accuracy of CTCs detection in gastric cancer. Table S2. The correspondence between numbers and the studies. [file 1471-2407-13-314-S1.zip › Supplementary Figure 6.tif]

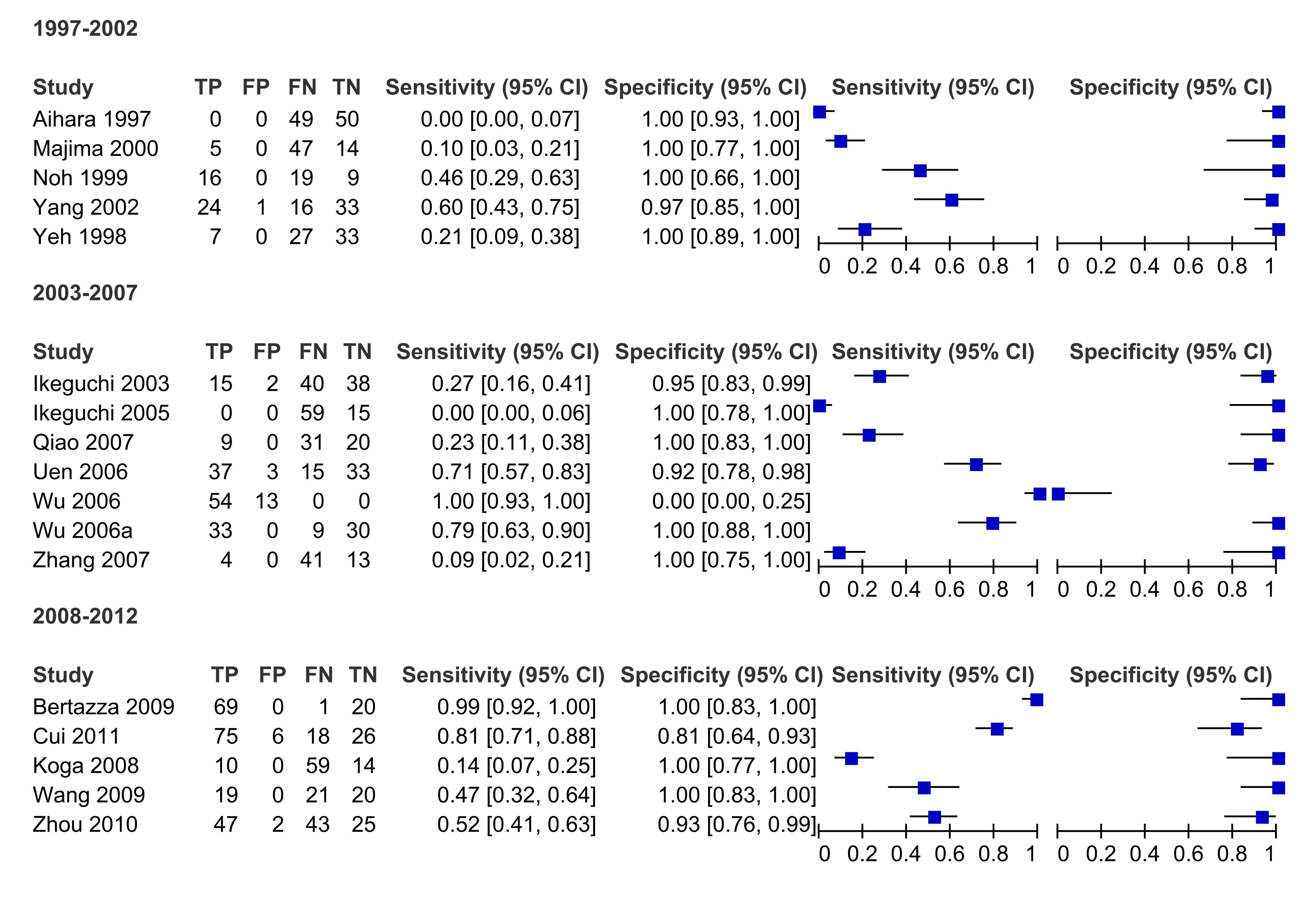

Supplement: Additional file 2: Figure S7 — Forest plots of sensitivity and specificity of CTCs detection in different published years among PCR-based group. [file 1471-2407-13-314-S2.tiff]

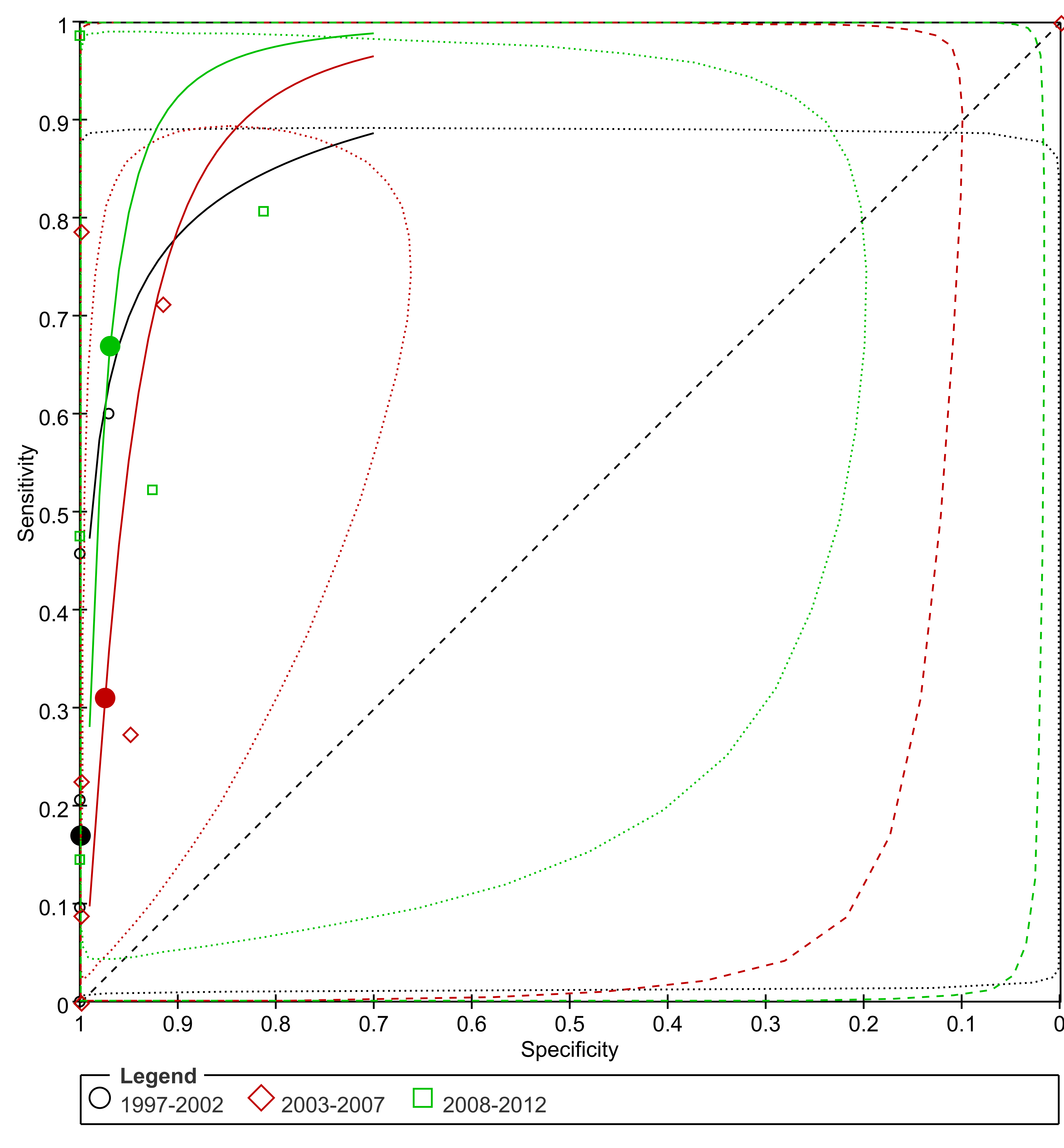

Supplement: Additional file 3: Figure S8 — Summary ROC plot of SEN and SPE of CTCs detection in different published years among PCR-based group. (Dotted ellipses around the spots represent the 95% CI around the summary estimates. The diamonds, rectangles and circles represent individual studies and size of the diamonds/rectangles/circles is proportional to the number of patients included in the study). [file 1471-2407-13-314-S3.tiff]
